# The anatomical technique of injection, dissection and colored segmentation of the venous system: Claude Gillot’s coloring technique

**DOI:** 10.1007/s12565-025-00907-5

**Published:** 2025-11-06

**Authors:** Jean Francois Uhl, Claude Gillot, José Ramon Mogorron Huerta, Manuel De Jesus Encarnacion Ramirez, Gervith Reyes Soto, Carlos Castillo Rangel, Vladimir Nikolenko, Nicola Montemurro

**Affiliations:** 1 UNESCO Chair in Digital Anatomy, Paris-Cité University, Paris, France; 2 Former Department of Anatomy, Descartes University, Paris, France; 3 Anatomy and Histology, Institute of Clinical Medicine named after N.V. Sklifosovskiy, Moscow, Russian Federation; 4 Department of Head and Neck, Unidad de Neurociencias, Instituto Nacional de Cancerología, INCAN, Universidad Nacional Autónoma de México (UNAM), Mexico City, Mexico; 5 Department of Neurosurgery, 1th of October Hospital, Mexico City, Mexico; 6 Human Anatomy and Histology Department, I.M. Sechenov First Moscow State Medical University (Sechenov University), Moscow, Russia; 7 Department of Neurosurgery, Azienda Ospedaliero Universitaria Pisana (AOUP), Pisa, Italy

**Keywords:** Cadaveric injection, Latex, Venous anatomy, Lower limb, Anatomical education

## Abstract

Injection of colored media remains pivotal for three‑dimensional appreciation of vascular anatomy since the pioneering work of Harvey, Ruysch and Swammerdam. Claude Gillot revived the approach for the study of the venous system by combining green‑latex infusion with post‑dissection vein painting (“colored segmentation”) to enhance anatomical education. To detail Gillot’s injection technique, evaluate its technical reliability, 400 fresh lower limbs (200 donors, mean age 75 years; Centre du Don des Corps, Paris) were irrigated with warm soapy water and injected via an ankle 19‑G butterfly into the great saphenous vein with filtered green latex (120–150 ml; 20 ml syringe; 20–30 kPa). Proximal femoral venous drainage prevented reflux. After 24 h polymerization the limbs were dissected; venous segments were painted according to a seven‑color palette. Patency, leakage and dissection time were recorded. Three exemplary specimens were photogrammetrically documented. Overall venous patency reached 93% with minimal segmental leakage (mean < 2 cm per limb). Dissection time per lower limb averaged ten hours. Gillot’s colored‑segmentation protocol provides a vivid, dependable and inexpensive platform for teaching and research in venous anatomy. Its flexibility and compatibility with digital capture surpass many contemporary embalming or silicone‑based perfusion techniques. Future work should integrate three‑dimensional models into virtual‑reality curricula and quantify learning outcomes.

## Introduction

The demonstration of the vascular pathways has been the foundation of anatomical science since William Harvey’s discovery of circulation in 1628. Seventeenth‑century innovators by the Dutch anatomists such as Frederik Ruysch and Jan Swammerdam perfected wax and mercury injections that immortalized fine vessels for display. Despite subsequent advances, contemporary medical curricula often deliver vascular anatomy as flat diagrams, leaving students struggling with spatial complexity.

Claude Gillot (Paris, 1970s–2000s) refined latex venous perfusion combined with post‑dissection “colored segmentation”, whereby each venous tributary is painted a distinct hue, producing a didactic three‑dimensional map (Uhl and Gillot 2012). The present study sets out to (1) describe the protocol in reproducible detail, (2) assess technical quality across multiple specimens and (3) showcase its educational value through three case dissections. This technique’s main innovation is the colored segmentation of each vein after identification, providing a unique educational tool for teaching and learning human anatomy.

### History of injection techniques in human anatomy

Injection into the vascular system is an ancient technique that was developed after William Harvey’s theory of blood circulation was established in the sixteenth century. Frideric Ruysch, an anatomist renowned for his arterial and venous injections, and J. Swammerdam, who was the first to use solidifying materials, were among the pioneers of this technique. Some landmarks in the history of vascular injections are shown in Table 1 (from Regis Orly 1998). Latex is a commonly used material for these injections. It is inexpensive, easy to use, non-toxic and water-soluble. It has low viscosity, which allows it to be easily injected into small vessels, and it becomes flexible once solidified, allowing the vessels to be manipulated during dissection without damaging them. Identification and cannulation of vessels: the common carotid arteries (CCA), internal jugular veins (IJV) and vertebral arteries (VA) are identified and cannulated using Foley catheters or intravenous tubing suitable for the diameter of the vessels.

**Table 1:** Landmarks in the history of vascular injection

Author	Date	Substance	Equipment
A. Gigliani (?)	1320	(?)	(?)
L. Da Vinci	1504	Wax	(?)
G.B. da Capri	1522	Warm water	Syringe
A. Vesale	1543	(?)	Siphon
W.F. Von Hiden	1615	(?)	Cannula linked to a bladder
J. Swammerdam	1672	Melted wax	Copper syringe
H. Homberg	1699	Lead, tin, bismuth	Pneumatic apparatus
R. Vieussens	1706	Saffron tinting	(?)
A.C. Thebesius	1708	Water, colored wax	(?)
Ronhaut	1718	Gelatin	(?)
F. Ruysch	1726	“materia ceracea”	(?)
G.A. Langguth	1746	(?)	Sipho anatomicus

(?), unknown

### Irrigation of vessels and applications

It is essential to remove blood clots and obstructions to allow correct injection of the latex. Irrigation is performed using a 60 cm^3^ syringe filled with warm water, starting with the bilateral CCAs, then the bilateral VAs, the dominant internal jugular vein and finally the non-dominant internal jugular vein. Irrigation is considered effective when clear water flows out of the opposite vessel. For arterial injections, the latex is injected into the arterial system while pinching the venous system to prevent reflux. The injection pressure must be sufficient to fill the vessels, but not too high to avoid damaging them. Otherwise, for venous injections, care must be taken to ensure that the injection pressure is sufficient to fill the vessels, but not too high to avoid damaging them.

Injected latex specimens can be used to study the microvascular anatomy of the brain, including the vessels of the cerebral white matter and the lenticulostriate artery, as well as for training in specific surgical approaches.

### Ethical considerations

The use of human specimens for these injections must comply with strict ethical considerations, particularly regarding the procurement of specimens and their use for study or teaching purposes. These techniques are essential for the study of vascular anatomy and can be adapted to the specific needs of each study or training program.

## Methods of Gillot’s technique (Uhl and Gillot 2012)

### Cadaver procurement & ethical approval

Two hundred non-embalmed donors (mean age 75 years) were provided by the Centre du Don des Corps under informed consent compliant with French Law 2022‑46 and the Declaration of Helsinki. Ethical approval was obtained. Bodies were refrigerated at 4 °C and utilized within seven days post‑mortem. Donors gave informed consent specifying the use of their bodies for teaching and research. Following completion of the dissections, all remains were managed respectfully and returned to the Centre du Don des Corps, which oversees collective cremation and burial at the designated memorial site in Paris.

### Vessel cannulation

**Fig. 1:**
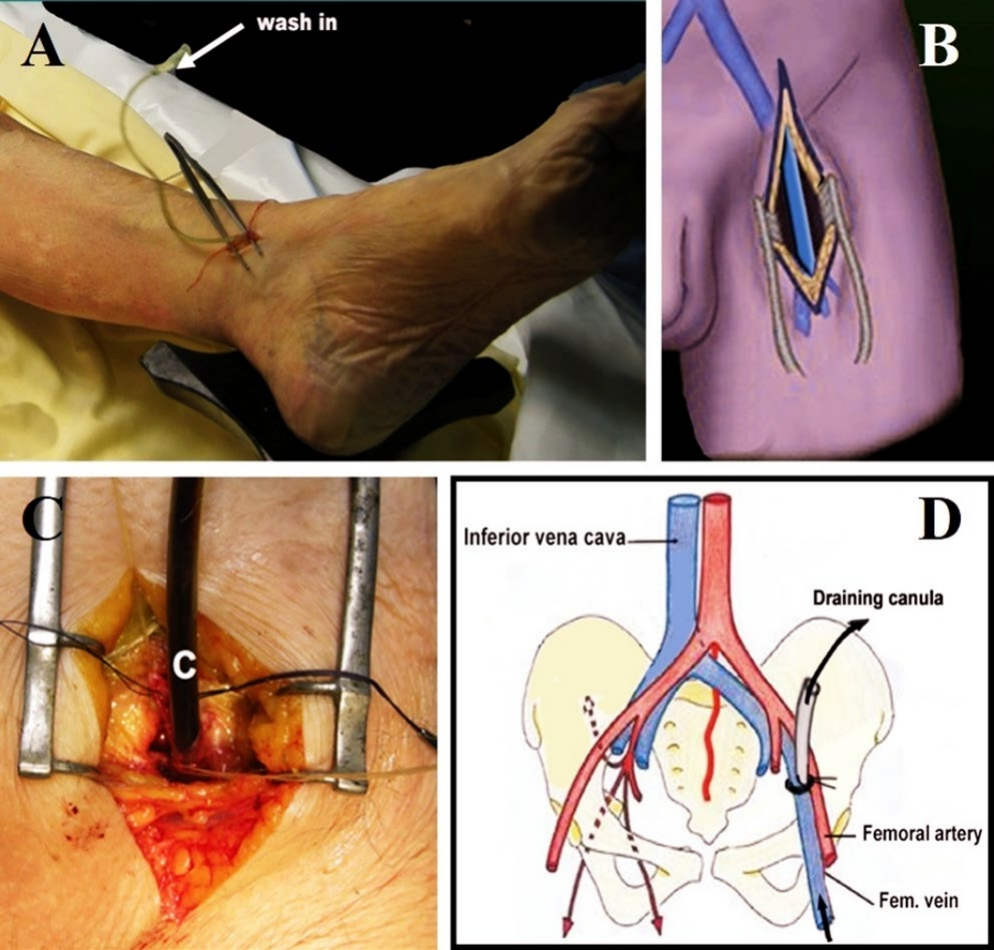
**A** Approach of the great saphenous vein (Gvs) at the ankle & introduction of a butterfly. **B** Common femoral vein approach through a vertical incision. **C** Insertion of a plastic cannula into the left common femoral. **D** Plastic cannula (C) to drain out the washing fluid

A three‑centimeter inframalleolar incision exposed the great saphenous vein. Then introduction of a butterfly 19-gauge catheter Terumo® (B) downwards, in the opposite direction of venous return (Fig. 1A). At the groin, common femoral vein is approach through vertical incision (Fig. 1B and 1C). A six‑millimeter plastic cannula (C) is secured in the common femoral vein for passive drainage (Fig. 1D).

**Fig. 2:**
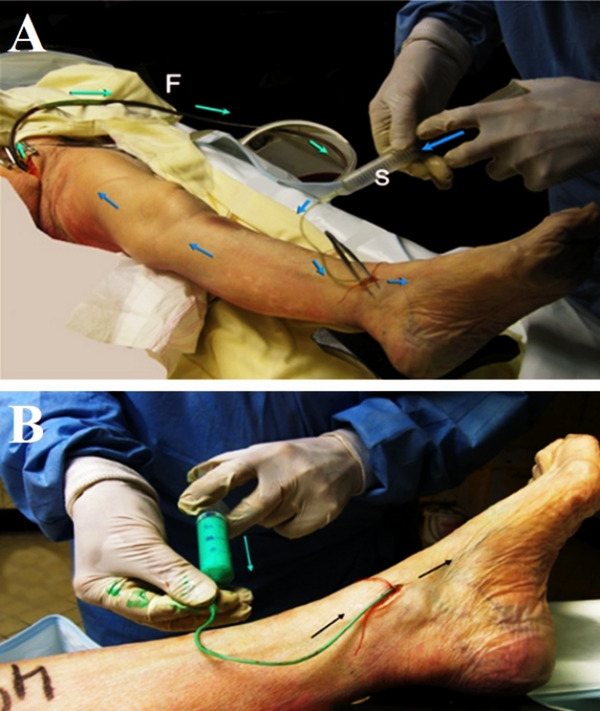
**A** The foot is irrigated with soapy water to remove the thrombi. **B** Filtered green latex is slowly injected through the saphenous catheter

Warm (38 °C) 0.1% detergent solution (soapy water) is then gently infused (Fig. 2A) using a 20 ml syringe (S). A massage of the leg and thigh muscles is performed to wash out remaining blood and thrombi. This procedure is repeated 2–4 times until the drained fluid is completely clear, assisted by distal‑to‑proximal limb massage. After removing the draining cannula and ligating the femoral vein, previously filtered commercial liquid green latex (Rothchild®, green) is slowly injected through the saphenous catheter using a 20 ml plastic syringe (Fig. 2B). The common femoral vein is then ligated and limbs rested at room temperature for twenty‑four hours to polymerize. Any leaking points are closed by simple compression.

During injection, occasional micro-ruptures of superficial venules or minor leakage (< 2 cm per segment) were observed in fewer than 7% of cases. These incidents did not compromise the global filling of the venous tree. When leakage occurred, it was corrected by localized compression or minimal cyanoacrylate sealing. No specimen was excluded for this reason, as even partially injected limbs retained valuable didactic and morphometric value.

### Dissection protocol

Anatomical dissection becomes possible one day after latex injection. After skin resection, dissection is performed in 3 steps.

**Fig. 3:**
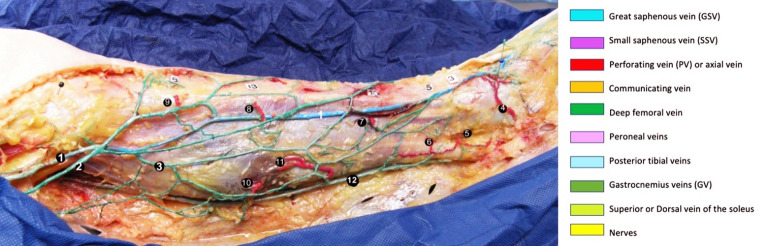
Skin ablation and dissection of the superficial plan of the leg with color codes used for dissections and drawings. 1 = GSV, 2 = Leg accessory of the GSV, 3 = Oblique communicating vein of the calf, 4 = Ankle PV, 5–6 = Lower PTVs, 7 = Higher PTV, 8 = Lower paratibial PV, 9 = Higher paratibial PV, 10 = Posterior PV of the calf, 11 = Central PV of the calf, 12 = SSV

- First, the superficial venous network is exposed by removing the subcutaneous fat. This step can take a long time in obese patients.
- Second, the perforating veins are identified and their position measured in relation to the bony landmarks. In this example in Fig. 3A, the posterior tibial PVs are located 3, 5 and 10 cm from the malleolar tip, respectively. The paratibial PVs are 8 and 13 cm from the knee joint space. Labels are placed next to each VP with their respective distances (white labels).
- Third, dissection of the deep system and muscular veins.

The time taken for dissection, patency and the non-injection segments are recorded.

### Colored segmentation palette and figure preparation

Post‑dissection, venous territories were painted according to Gillot’s multicolored palette (Fig. 3B) used for dissection and drawings. High‑resolution (300 dpi) DSLR photographs were captured before and after painting against a neutral background with scale bars. Figures were prepared in Adobe Photoshop; placeholders are indicated in the manuscript and high‑resolution pictures accompany this submission.

## Results

### Global quality of injection

Ninety‑three percent of identifiable venous segments were fully patent; incomplete filling was limited to perforators under one millimeter. Segmental leakage averaged less than two centimeters per limb and was controlled with direct compression. No arterial reflux occurred. Mean preparation time (irrigation plus injection) was fifty‑five ± eight minutes. Average dissection time per lower limb was ten hours, varying with adipose content and operator experience. While such duration may constrain reproducibility in short teaching sessions, segmental dissection can be distributed among student teams or focused on key venous territories to optimize instructional time. Our findings suggest that a two-day modular approach preserves educational value while maintaining procedural feasibility in most anatomy laboratories.

### Representative case studies

**Fig. 4:**
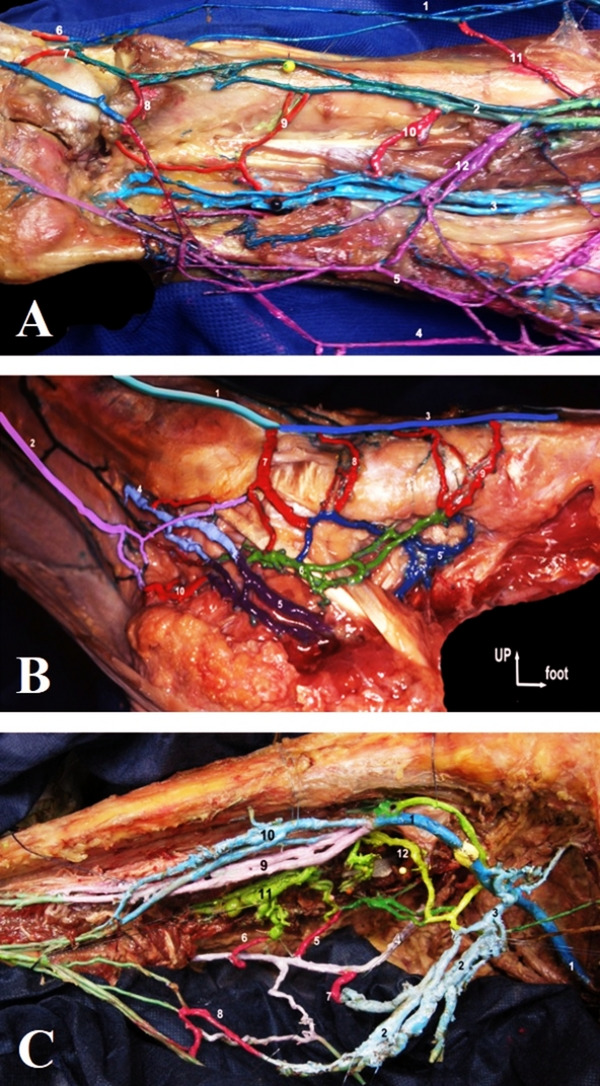
**A** Anatomical dissection of a left leg (lateral view). This unique final result by C. Gillot was possible only after having removed the fibular bone. Dorsal perforators of the ankle (6,7) give origin to the anterior tibial (1, in dark blue) and peroneal veins (2, in green) respectively 3 = posterior tibial veins (in light blue). 4 = Achillean tributary of the SSV 5 = SSV 8 = Ankle dorsal peroneal. Transverse deep communicating veins are displayed in red, connecting PTVs to peroneal veins (9,10) and peroneal veins to anterior tibial veins (11). A perforator (12) originated from the SSV, drains into the peroneal veins (2). **B** Medial anatomical/functional unit of the foot (dissection of a left foot, medial view). 1 = GSV initial segment over the medial malleolus, 2 = Achillean tributary of the small saphenous vein, 3 = medial marginal vein, 4 = posterior tibial veins, 5 = lateral plantar veins, 6 = medial plantar veins, 7 = inframalleolar PV, 8 = navicular PV, 9 = cuneiform PV, 10 = calcaneal PV. **C** Calf PVs and connections to gastrocnemius veins, soleal veins and SSV. 1 = popliteal vein, 2 = medial GV, 3 = medial common gastrocnemius venous trunk, 4 = SSV, 5–6 = soleal PVs, 7 = central calf PV, 8 = inferior, polar or Gillot´s calf PV 10 PT veins, 9 = peroneal veins, 11 = Lateral soleal veins, 12 = dorsal soleal vein

- Case 1 highlighted axial deep interconnections after fibula removal (Fig. 4A);
- Case 2 mapped the medial foot functional unit (Fig. 4B);
- Case 3 exposed soleal–gastrocnemius–SSV perforators (Fig. 4C).

All networks were clearly differentiated by color, facilitating landmark recognition and student quizzes.

## Discussion

Latex injection remains a cornerstone of anatomical specimen preparation owing to its low viscosity, flexibility and cost. Compared with Thiel embalming, which preserves tissue suppleness but can dilute pigments, Gillot’s protocol (Uhl and Gillot 2012) maintains vivid color without toxic fumes. Silicone perfusion yields high contrast but is costly and time‑consuming, whereas ethanol‑glycerin fixation demands closed‑loop perfusion and carries higher biohazard risks. Salt‑saturation cadavers stiffen over time, limiting dissection. Various venous mapping accuracy, innovations in injection media for anatomical research, techniques of injection, pressure controls, Quantitative evaluation of patency, and optimizations were reported in the literature (Reyes Soto et al. 2025; Gupta and Kohli 2023; Doomernik et al. 2016; Clark and Parsons 2023; Bayne and Murray 2020; Cole 1921; Martínez et al. 2020; Durongphan et al. 2022; Hammer et al. 2015; Hammer 2022; Smith et al. 2023; Thakur et al. 2012; Buehler et al. 2010; Calgari et al. 2021; Eguchi et al. 2024; Felder et al. 2021; Franco et al. 2015; Hasegawa et al. 2021; Iqbal et al. 2020; Janssen et al. 2024; Kelley et al. 2022; Nakata et al. 2021; O’Neill and McGrath  2022; Panneerselvam et al. 2024; Quinlan et al. 2023; Reddy et al. 2022; Tanaka et al. 2020; Valls et al. 2024; Young and Armitage 2021). Table 2 shows the comparison of the injection techniques. Gillot’s method is limited by the need for fresh cadavers and a twenty‑four‑hour curing delay. Future integration with three‑dimensional photogrammetry (Lin and Hsu 2023) and virtual‑reality simulators (Encarnacion et al. 2023; Bianchi et al. 2024) will broaden accessibility. Quantitative research should correlate injection success with donor age, post‑mortem interval and intraluminal pressure. While the future looks bright with the integration of 3D and VR, a few words on cost–benefit analysis in comparison to contemporary digital-only instruction (without cadavers) would be beneficial. The requirements for fresh cadavers, time-consuming dissections, and potential variability based on injector experience should be extended. Although 3D visualization and virtual-reality (VR) simulators have transformed anatomical education (Carbone et al. 2025; Montemurro et al. 2022; De Jesus et al. 2024; Autelitano et al. 2024; Ramirez et al. 2023), the cost of advanced digital setups often exceeds that of maintaining a small fresh-cadaveric unit (Panneerselvam et al. 2024; Quinlan et al 2023; Encarnacion et al. 2024). The Gillot protocol requires minimal consumables—latex (€ 2–3 per specimen) and detergent—making it one of the most cost-effective models for vascular anatomy. In contrast, commercial VR licenses can exceed € 20.000 annually. Nevertheless, the requirement for fresh cadavers, specialized refrigeration, and experienced injectors introduces variability in outcomes and limits universal adoption. From a cost–benefit perspective, the hybrid model (combining low-cost cadaveric segmentation with digital 3D rendering) offers the highest pedagogical return, merging tactile realism with scalable access.

**Table 2:** Comparison of physical and logistical properties of common injection/embalming media

Medium	Cost (€)	Viscosity (cP)	Cure time	Toxicity	Color stability	Flexibility
Latex	Low	30–50	24 h	Low	High	High
Silicone	High	500–1000	48 to 72 h	Low	Very high	Moderate
Thiel	Moderate	-	-	Moderate	Moderate	Very high
Ethanol-glycerin	Low-moderate	-	-	High	Moderate	High
Salt-saturation	Low	-	-	Low	Low	Low

## Conclusions

Gillot’s colored‑latex venous injection technique on fresh cadavers produces highly patent, vividly segmented specimens that significantly enhance comprehension of lower‑limb venous anatomy while remaining economical and ethically sustainable. Its adaptability to modern digital platforms makes it a valuable addition to contemporary anatomical education and research.

## Data Availability

No datasets were generated or analysed during the current study.
